# Mapping the medical status of patients in a dental school: adapting dental curricula to demographic change - a cross-sectional study

**DOI:** 10.1186/s12909-025-08180-w

**Published:** 2025-11-06

**Authors:** Marco M. Herz, Michael Scharl, Jana Ripperger, Diana Wolff, Valentin Bartha

**Affiliations:** 1 Department of Conservative Dentistry, University of Tuebingen, Osianderstr. 2-8, Tuebingen, 72070 Germany; 2 Private Practice, Boeblingen, Germany; 3 Department for Conservative Dentistry, Heidelberg University, Im Neuenheimer Feld 400, Heidelberg, 69120 Germany

**Keywords:** Dental school, Dental curriculum, Multimorbid patients, Medication, Dental education

## Abstract

**Background:**

Medical assessment of patients treated by dental school students with regard to medical history, medication use and allergies to determine potential medical risks of the changing population structure and to develop implications for future curriculum design.

**Methods:**

A cross-sectional study was conducted to assess the medical records of patients, treated between November 2020 and October 2021, for demographic data (age, sex), allergies, systemic disorders, existing diseases, and medication use. Diseases were categorized according to the International Classification of Diseases (ICD), while medication was classified based on the Anatomical Therapeutic Chemical (ATC) Classification. A statistical analysis of the correlations between patient characteristics and prevalence data was performed.

**Results:**

Data of 297 participants were analysed, including 142 women (mean age 55.51 ± 14.9 yrs) and 155 men (54.91 ± 15.52 yrs). Systemic diseases were present in 189 individuals (63.6%), 178 (60.1%) were taking medication, and 138 (46.5%) had at least one allergy. Polypharmacy (≥ 3 medications) was observed in 28% of participants (mean age 62.4 years). Medication use and disease burden increased significantly with age (e.g., cardiovascular medication: OR = 1.09 per year; 95% CI: 1.07–1.12; *p* < 0.001). A statistically significant sex difference was observed for hormonal medication (ATC H: 68% female vs. 32% male; *p* = 0.0012).

**Conclusion:**

The observed advanced age profile of the patients and its correlation with the prevalence of systemic diseases, medication use, and allergies demonstrated the medical complexity of dental patients care. These observations emphasize the importance of providing undergraduates but also postgraduates with a more comprehensive medical education to prepare them to effectively treat medically complex patients.

**Supplementary Information:**

The online version contains supplementary material available at 10.1186/s12909-025-08180-w.

## Background

 Human life expectancy has risen significantly in recent decades [1, 2]. This might be attributed to the high standard of living, the significant improvement in people’s working conditions and, above all, the constant new medical and pharmacological developments in the treatment of diseases [3]. Nevertheless, these increasingly elderly patients are not necessarily very healthy, and some studies show that the patient morbidity will increase significantly. In particular, the number of patients who are diagnosed with several diseases at the same time and therefore have a much more complex morbidity, will increase significantly as well, especially in the distant future [4–6]. Additionally, systemic diseases such as diabetes and cardiovascular disease can increase the risk of complications during dental procedures. However, patients, including those with multiple illnesses, can now counteract their limitations with new drugs and medical devices. As a result, they appear to look and often feel healthy and can participate in life without restrictions [7–11]. As they are often only treated symptomatically, these patients are not cured of their diseases and remain at risk due to their medical history. Furthermore, certain medications, including anticoagulants and antiresorptives, can continue to present notable risks during dental procedures [12–14]. Furthermore, these patients are constantly exposed to an increased risk of medical incidents and emergencies [15, 16]. Some studies in the past have shown that incidents can occur during dental treatment and that even resuscitation measures may even have to be taken [17–20]. Multimorbid and elderly patients therefore require special attention, as even routine dental treatments can be physically demanding and may involve the use of local anaesthesia and other medications that may interact with previously administered medications. Furthermore, the interaction with non-communicable diseases such as diabetes underlines the importance of addressing common risk factors [21]. In this context, Lamster and Eaves also suggested expanding the role of dental practices to include primary healthcare activities such as screening for hypertension and diabetes [22]. Possible activities in this area could also include measures to stop smoking and combat obesity. These ideas illustrate the potential of dental practices to improve overall health outcomes [22].

According to the licensing regulations for dentists, dental students should treat patients independently and on their own responsibility at an early stage in the dental curriculum [23]. As inexperienced practitioners, dental students are the first to be encountered with possible incidents. They must be able to correctly classify the medical history of a patient in connection with the planned dental treatment and assess the possible effects of general diseases, allergies and medications. Ideally, students should already have the relevant background knowledge on these topics and the corresponding teaching modules should take place prior to the first patient treatment. Although this would be desirable, it is not guaranteed due to the wide range of topics in the curriculum. In addition, since the licensing regulations only stipulate which subjects are taught in which semester and to what extent in the curriculum, and which examinations are to be taken in the respective subject, great differences may certainly be expected from university to university. There are some studies that evaluate the typical medical profile of patients treated in dental schools [21, 22, 24–26]. Studies reported the prevalence of systemic diseases in dental patients, with 41% having at least one systemic condition such as hypertension or diabetes [24]. Medically compromised patients often required modified or adjusted treatment protocols, including sedation or antibiotic prophylaxis [25]. Al-Habib et al. demonstrated that a greater awareness of the oral-systemic links among physicians also had an impact on the referral of patients to dentists [26]. Fernández-Feijoo, J. et al. found that there is a high prevalence of medical conditions and patients with polypharmacy among people seeking dental care [27]. A study group led by Humbert et al. investigated and identified a large number of internal diseases, disorders and syndromes that dental students are confronted with during their training in order to be able to make recommendations for teaching so that dentists later feel better prepared for possible emergencies in practice [28]. Furthermore, Al-Bayaty used a questionnaire to determine 303 medical conditions in 571 patients at a dental school and described this knowledge as an essential gain to the dental curriculum [29].

In recent times, increasing attention has been given to the influence of aging and polypharmacy in geriatric patients on oral health, dental therapeutic approaches, and the risk of pharmacological interactions [30–34]. In addition to these studies, there are also national and international articles and publications by professional societies or organisations that address this topic [35, 36].

Despite these insights, further research is needed to address the challenges associated with multimorbid and systemically ill patients, aiming to provide a broader scientific background for the development of future educational curricula. Nevertheless, some gerodontological societies have already taken up this topic, including for the training of students, and offer curricular programmes on this subject [37, 38].

In recent years, there has been a noticeable decline in the number of patients attending student courses, making patient recruitment increasingly challenging. At the same time, anamnesis and treatment-related case discussions with students have become more complex and time-consuming.

Therefore, the aim of this study was to get a general overview of systemic health condition, medication use, and allergies of patients treated in the department of conservative dentistry at a University Hospital in the south of Germany. The primary outcome of the study was a profiling of the patients seen within the dental curriculum regarding prevalence of diseases, allergies and the consumption of medication. As a secondary outcome, the disease, medication and allergy burden per patient, defined as number of diseases, medications used and allergies present, was assessed and the influence of age and sex on these variables was analysed. Finally, the findings were used to derive recommendations for adapting or updating the dental curriculum.

## Methods

### Study-design and setting

The study was conducted in accordance with the Declaration of Helsinki. It was approved by the Ethics Committee of the University of Tübingen (reference number 120/2020B02) and registered in the German Clinical Trials Register (URL: https://www.drks.de; ID: DRKS00029310). All patients had provided informed consent for the use of anonymized health records for research purposes, as approved by the local ethics committee.

The report of the study followed the STROBE guidelines.

### Participants

There are different ways for patients to participate in the student course. In principle, the University Dental Clinic is open to all patients for dental treatment. Since the dental clinic has little influence on the influx of patients, the student courses are dependent on the number of patients who (A) visit the dental clinic and (B) request treatment in the student courses.

However, certain criteria are checked by a dental supervisor to determine whether treatment is possible in a student course.

Inclusion criteria for treatment by undergraduates:

- agreement on treatment by dental students.

Exclusion criteria for treatment by undergraduates:

- infectious disease (e.g., HIV, Hepatitis C).
- life threatening disease (e.g., immunosuppression, leukaemia).

The patients therefore had to give two different consents, a verbal but documented consent for general treatment by students and an informed consent for specific participation in this study.

### Variables

Based on the medical records of the participating patients, the following entries were recorded:

- demographic data (age and sex).
- allergies.
- systemic or chronic disorders and existing diseases.
- medication intake.

On admission to the dental clinic, each patient was given a medical history form to complete, which was checked for completeness by the examining dentist and supplemented with additional questions for clarification if necessary.

### Data sources and quantitative variables

For analysis and better comparability with other studies and population statistics, the patients were divided into three age groups: ≤ 44 years, 45–64 years, and 65 years and older.

The disorders were clustered according to the ICD-International Classification of Diseases [39]. All drugs and medications recorded were assigned to the different areas of application or drug groups based on the Anatomical Therapeutic Chemical (ATC) Classification [40]. The intolerances and allergies reported by the patients were categorized on the basis of the article by Kleine-Tebbe et al. [41]. Since all participants were interviewed personally, there are no missing data—unless patients chose not to provide truthful responses.

### Bias

During the study period, all treating students were instructed by the study supervisor in a kick-off event at the beginning of each semester by means of a lecture on how they should care for the patients with regard to the study content. The students should assist the participants with questions about consent and data protection, critically review the medical history forms for completeness before starting treatment and, if necessary, ask specific questions to ensure that the answers are noted accurately.

These forms had to be re-checked by the treating student on each treatment day to ensure that they were up to date and complete. If the last treatment was more than 6 months ago, the patient had to fill out a completely new form before the next treatment.

### Study size

**Fig. 1:**
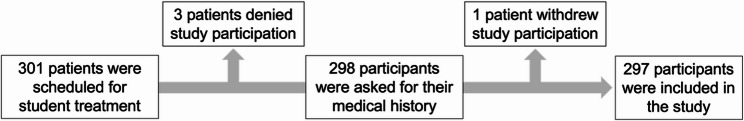
Flowchart for selecting participants and obtaining medical history

For this study, all patients, who were treated by students in the Department of Conservative Dentistry over a period of one year from November 2020 to October 2021 (two semesters) and who gave their informed consent to participate were included (Fig. 1). No a priori sample size calculation or power analysis was conducted, as the primary aim was to include the full eligible population in order to describe the medical profiles of patients treated in the student dental curriculum rather than to test a predefined hypothesis.

### Statistical methods

Descriptive statistics, including percentages, were used to summarize patient demographics, disease prevalence, medication use, and allergies. Categorical variables were analysed using the chi-square test in order to assess the differences between the groups (e.g., age categories and sex). Continuous variables showed a significant deviation from normality according to the Anderson-Darling test (all *p* < 0.0001). Representative values include: age (A² = 2.54), number of diagnoses (A² = 16.25), and number of medications (A² = 23.42). Consequently, non-parametric statistical methods were used consistently. Hence, continuous data were analysed using the Wilcoxon rank-sum test. Binomial regression analysis was performed to evaluate associations between independent variables (e.g., age and sex) and specific outcomes (e.g., prevalence of diseases or medication intake), with odds ratios (ORs) reported to quantify the strength of these associations. Model fit was evaluated using the Akaike Information Criterion (AICc), the Bayesian Information Criterion (BIC), and McFadden’s pseudo *R*².All statistical analyses were performed using the JMP^®^ (JMP 16.0, SAS Institute GmbH, Heidelberg, Germany), with a significance level set at *p* < 0.05.

## Results

The results are presented in accordance with the study objectives, beginning with descriptive profiling of the patient population (primary outcome), followed by analyses of disease and medication burden and their associations with demographic factors (secondary outcome).

### Participant demographics

A total of 297 patients, 142 women (47.8%) and 155 men (52.2%), treated during the two semesters and final examinations met the inclusion criteria and were included in the analysis. 76 participants (25.6%) were smokers. Of these, 35 participants smoked up to 10 cigarettes per day, and 39 smoked more than 10 cigarettes per day; no information was available for 2 patients. 73 patients (24.6%) stated that they consumed alcohol, 57 of them described their consumption as rare, 9 as frequent, and 7 did not provide precise information on frequency. The youngest patient was 14yrs old, the oldest 85yrs. The mean age was 55yrs. 65 patients (21.9%) were ≤ 44yrs old, 143 (48.1%) were aged between 45-64yrs, and 89 (30.0%) ≥ 65yrs old (Table 1).

**Table 1:** Disease status and medication intake of participants

Participants’ categories	Sex	Participants by age group (*n*/%)			Total (*n*/%)
		≤ 44yrs	45-64yrs	≥ 65yrs	
No general disease	F	17/5.7	21/7.1	8/2.7	46/15.2
	M	23/7.7	35/11.8	4/1.4	62/20.7
	T	40/13.4	56/18.9	12/4.1	108/36.5
General diseases	F	12/4.0	50/16.8	34/11.5	96/32.5
	M	13/4.4	41/13.8	39/13.1	93/31.5
	T	25/8.4	91/30.6	73/24.6	189/63.6
Not taking any medication	F	18/6.0	28/9.4	9/3.0	54/18.2
	M	21/7.1	35/11.8	8/2.7	64/21.6
	T	39/13.1	63/21.2	17/5.7	119/39.8
Taking medication	F	11/3.7	43/14.5	33/11.1	87/29.3
	M	15/5.1	41/13.8	35/11.8	91/30.8
	T	26/8.8	84/28.3	68/22.9	178/60.1

*Abbreviations*: *F *female, *M *male, *T *total

### Distribution of systemic diseases and medication use throughout all age groups

Overall, there are more participants with disorders (*n* = 189/63.6%) than without disorders (*n* = 108/36.5%). A total of 518 diseases were recorded in the 189 participants. The prevalence of systemic diseases varied across the age groups. Within the age group ≤ 44yrs there was a higher proportion of healthy participants (13.4%) compared to participants with disorders (8.4%). In the older age groups, the proportion of participants with health conditions predominated: in the age group 45-64yrs there were 30.6% with chronic diseases and 18.9% healthy patients. Similarly, among participants ≥ 65yrs, there were 24.6% participants with disorders compared to 4.1% healthy patients (Table 1). These differences were statistically significant (*p* < 0.001), with no significant effect of sex.

Similarly, 178 participants (60.1%) took medication in comparison to 119 (40.0%) who did not. These 178 patients took a total of 571 medications, which corresponds to an average of 3.2 medications per affected patient, and 1.9 for all participants. Medication was more prevalent in older age groups. Within the age group ≤ 44yrs, more participants took no medication (13.1%) than those who did (8.8%). In contrast, in the age group 45–64yrs was a higher proportion of participants that took medication (28.3%) compared to those who did not (21.2%). This ratio was even more pronounced in the age group the ≥ 65yrs with 22.9% taking medications and 5.7% taking no medications (Table 1). Again, these differences were statistically significant (*p* < 0.001) without significant influence of sex.

### Age-category related patterns in disease prevalence and medication use

No statistically significant patterns of illness or medication use were found in the under 45 age group. In contrast, logistic regression analysis revealed a statistically significant positive correlation among 45–64-year-olds for diseases as a whole (*p* = 0.006), for ICD-Codes XVIII (Symptoms, signs and abnormal clinical and laboratory findings, not elsewhere classified) (*p* = 0.0499), and for XXI (Factors influencing health status and contact with health services) (*p* = 0.009). Moreover, a statistically significant positive correlation was found for the following diseases in the ≥ 65yrs-group: diseases of the eye and adnexa (*p* = 0.0421), diseases of the blood/blood-forming organs, and certain disorders involving the immune mechanism (*p* = 0.0258), and diseases of the circulatory system (*p* = 0.0312). There is a statistically significant association (*p* = 0.0473) with medications for the alimentary system and metabolism by age at participation.

### Overall prevalence of systemic diseases

Diseases of the circulatory system were most prevalent (*n*=170), followed by endocrine, nutritional and metabolic diseases (*n*=84) and factors influencing health status and contact with health services (*n*=54). The latter includes conditions following stent insertion, chemotherapy or radiotherapy. Age was significantly associated with several ICD-10 disease categories, including circulatory (IX), endocrine (IV), musculoskeletal (XIII), neoplastic (II), and mental disorders (V). For example, the model for circulatory diseases showed an OR of 1.07 (95% CI: 1.054–1.100; *p* < 0.001), with McFadden’s *R*² = 0.16, AICc = 345.5, BIC = 352.8. Neoplasms showed an OR of 1.04 (95% CI: 1.006–1.087; *p* = 0.019; *R*² = 0.043), and notably mental disorders were negatively associated with age (OR = 0.97; 95% CI: 0.945–0.999; *p* = 0.049; *R*² = 0.025), though model fit was low for this category (S-Table 1).

Furthermore, a small but significant sex-association was found for endocrine diseases (68% female vs. 32% male; Cramér’s V = 0.13). For respiratory diseases the association was borderline significant with a very small effect (V = 0.11). No other ICD-10 chapters showed statistically relevant sex differences (all *p* > 0.05, V < 0.10). (Table 2). S-Table 2 summarizes sex frequencies for variables with significant sex associations.

**Table 2:** Number of diseases assigned to ICD categories. Odds ratio per year of age for suffering from diseases of each category using logistic regression; *p*-values from the chi-square tests for sex differences within each category

ICD-10-Chapter		Total		Effect of age					Effect of sex
		*N*	%	Esti-mate	*p*-Value	OR	LCI	UCI	*p*-Value
IX	Diseases of the circulatory system	170	57.2	0.074	< 0.001*	1.07	1.054	1.100	0.44
IV	Endocrine, nutritional, and metabolic diseases	84	28.3	0.043	< 0.001*	1.04	1023	1065	0.02*
XXI	Factors influencing health status and contact with health services	54	18.2	0.070	< 0.001*	1.07	1.038	1.107	0.81
XIII	Diseases of the musculoskeletal system and connective tissue	42	14.1	0.021	0.008*	1.04	0.979	1.064	0,1
X	Diseases of the respiratory system	34	11.4	0.018	0.18	1.02	0.991	1.045	0.049*
V	Mental and behavioural disorders	24	8.1	−0.028	0.049*	0.97	0.945	0.999	0.07
XI	Diseases of the digestive system	21	7.1	0.020	0.24	1.02	0.986	1.056	0.24
II	Neoplasms	20	6.7	0.043	0.019*	1.04	1.006	1.087	0.26
III	Diseases of the blood/blood-forming organs, and certain disorders involving the immune mechanisms	16	5.4	0.036	0.07	1.03	0.997	1077	0.49
VI	Diseases of the nervous system	14	4.7	0.016	0.42	1.01	0.977	1056	0.90
XVIII	Symptoms, signs and abnormal clinical and laboratory findings, not elsewhere classified	13	4.4	0.021	0.32	1.02	0.979	1.064	0.05
XIV	Diseases of the genitourinary System	9	3.0	0.028	0.31	1.03	0.973	1.088	0.79
VII	Diseases of the eye and adnexa	7	2.4	0.097	0.024*	1.10	1012	1200	0.35
XII	Diseases of the skin and subcutaneous tissue	5	1.7	0.021	0.52	1.02	0.958	1.088	0.58
	Others	5	1.6		-	-			-

*Abbreviations*: *ICD* international classification of diseases, *OR* odds ratio

*statistically significant; Percentages refer to the number of recorded diseases per category relative to the total number of patients; multiple entries per patient possible

### Overall prevalence of medication intake

A total of 178 participants took 571 medications, with an average of 3.2 medications per patient. Medications for the cardiovascular system (*n* = 214, classification C) were used most frequently, followed by medications that affect the digestive tract and metabolism (*n* = 82, classification A), including oral antidiabetics such as metformin or proton pump inhibitors such as omeprazole. In third place were medications that affect the blood and blood forming organs (*n* = 65, classification B, in particular antiplatelet agents such as medications containing acetylsalicylic acid. This was followed by medication for the nervous system (*n* = 60, classification N), the hormonal system (*n* = 54, classification H), and the musculoskeletal system (*n* = 28, classification M). Medication for C, A, B, H, and M showed a statistically significant relationship to age using logistic regression (S-Table 3). For these significant associations with age, model fit was acceptable with AICc values ranging from 176.1 to 303.7 and McFadden’s R² between 0.02 and 0.19. Moreover, the chi-square test revealed a statistically significant association between sex and the use of ATC category H medications (χ²(1) = 10.46, *p* = 0.0012), with 68% of users being female and 32% male. The effect size, expressed as Cramér’s V, was 0.134, indicating a small to moderate association. (Table 3). S-Table 2 summarizes sex frequencies for variables with significant sex associations.

**Table 3:** Number of medications classified according to ATC categories. Odds ratio per year of age for medication intake per category using logistic regression, and *p*-values from chi-square tests for sex differences within each category

ATC classification		Total		Effect of age					Effect of sex
		*n*	%	Estimate	*p*-value	OR	LCI	UCI	*p*-value
C	Cardiovascular system	214	72.1	0.088	< 0.001*	1.09	1.065	1.121	0.39
A	Alimentary tract and metabolism	82	27.6	0.039	< 0.001*	1.04	1.016	1.063	0.34
B	Blood and blood forming organs	65	21.9	0.092	< 0.001*	1.10	1.063	1.130	0.27
N	Nervous system	60	20.2	−0.002	0.83	0.99	0.976	1.019	0.33
H	Systemic hormonal preparations, excluding sex hormones and insulins	54	18.2	0.026	0.016*	1.03	1.004	1.049	0.001*
M	Musculo-skeletal system	28	9.4	0.045	0.005*	1.05	1.013	1.080	0.40
L	Antineoplastic and immunomodulating agents	17	5.7	0.001	0.91	1.00	0.969	1.036	0.23
R	Respiratory system	17	5.7	0.019	0.28	1.02	0.984	1.055	0.66
G	Genito urinary system and sex hormones	16	5.4	0.019	0.31	1.02	0.981	1.059	0.47
J	Anti-infective for systemic use	5	1.7	−0.063	0.09	0.94	0.871	1.010	0.61
S	Sensory organs	5	1.7	0.070	0.09	1.07	0.989	1.164	0.58
	Others	8	2.7		-	-			-

*Abbreviations*: *ATC* anatomic therapeutic chemical, *OR* odds ratio, *LCI* lower confidence interval, *UCI* upper confidence interval

*statistically significant; Percentages refer to the number of recorded diseases per category relative to the total number of patients; multiple entries per patient possible

### Disease and medication burden by patient age and sex

Approximately two thirds of all patients reported at least one disease (mean age 59.5 ± 13.8 yrs), while 43% stated that they suffered from 3 or more diseases (mean age 62.4 ± 12.2 yrs) (Table 4). There was no statistically significant gender-specific difference using chi-squared test. The mean age of patients with one or two diseases was 52.5 ± 15.3 years, while that of patients with three or more diseases was 62.4 ± 12.1 years. Similarly, the mean age of patients taking three or more medications was 64.4 ± 12.8 years, which was again considerably higher than that of patients taking 1 and 2 medications (51.5 ± 14.5 years).

**Table 4:** Characteristics of patients with one or more diseases and one or more medication taken

No. of diseases	Diseases						No. of medications taken	Medication					
	Female		Male		Total			Female		Male		Total	
	*n* (%)	mean age	*n* (%)	mean age	*n* (%)	mean age		*n* (%)	mean age	*n* (%)	mean age	*n* (%)	mean age
1	27 (14.3)	51.9 ± 13.8	32 (16.9)	55.8 ± 14.2	59 (31.2)	54.0 ± 14.0	1	29 (16.3)	50.0 ± 14.2	32 (18.0)	52.7 ± 15.0	61 (34.3)	51.4 ± 14.6
2	28 (14.8)	60.9 ± 11.9	22 (11.6)	62.1 ± 17.2	50 (26.5)	61.5 ± 14.3	2	19 (10.7)	58.4 ± 9.7	14 (7.9)	59.0 ± 14.8	33 (18.5)	58.7 ± 11.9
3	14 (7.4)	60.1 ± 12.8	14 (7.4)	61.8 ± 12.1	28 (14.8)	60.9 ± 12.3	3	11 (6.2)	64.5 ± 11.7	13 (7.3)	68.2 ± 11.2	24 (13.5)	66.5 ± 11.3
4	11 (5.8)	60.0 ± 9.8	10 (5.3)	61.6 ± 8.8	21 (11.1)	60.7 ± 9.1	4	3 (1.7)	71.3 ± 3.8	7 (3.9)	66.7 ± 12.9	10 (5.6)	68.1 ± 10.9
5	8 (4.2)	63.3 ± 11.5	9 (4.8)	59.4 ± 19.3	17 (9.0)	61.2 ± 15.7	5	12 (6.7)	66.3 ± 8.0	10 (5.6)	50.1 ± 16.7	22 (12.4)	58.9 ± 14.9
6–10	7 (3.7)	69 ± 12.9	5 (2.6)	70.8 ± 7.0	12 (6.3)	69.8 ± 10.5	6–10	11 (6.2)	65.5 ± 14.0	15 (8.4)	66.1 ± 11.9	26 (14.6)	65.9 ± 12.5
> 10	1 (0.5)	64.0	1 (0.5)	68.0	2 (1.1)	66.0 ± 2.8	> 10	2 (1.1)	62.5 ± 7.8	0 (0.0)	-	2 (1.1)	62.5 ± 7.8

**Fig. 2:**
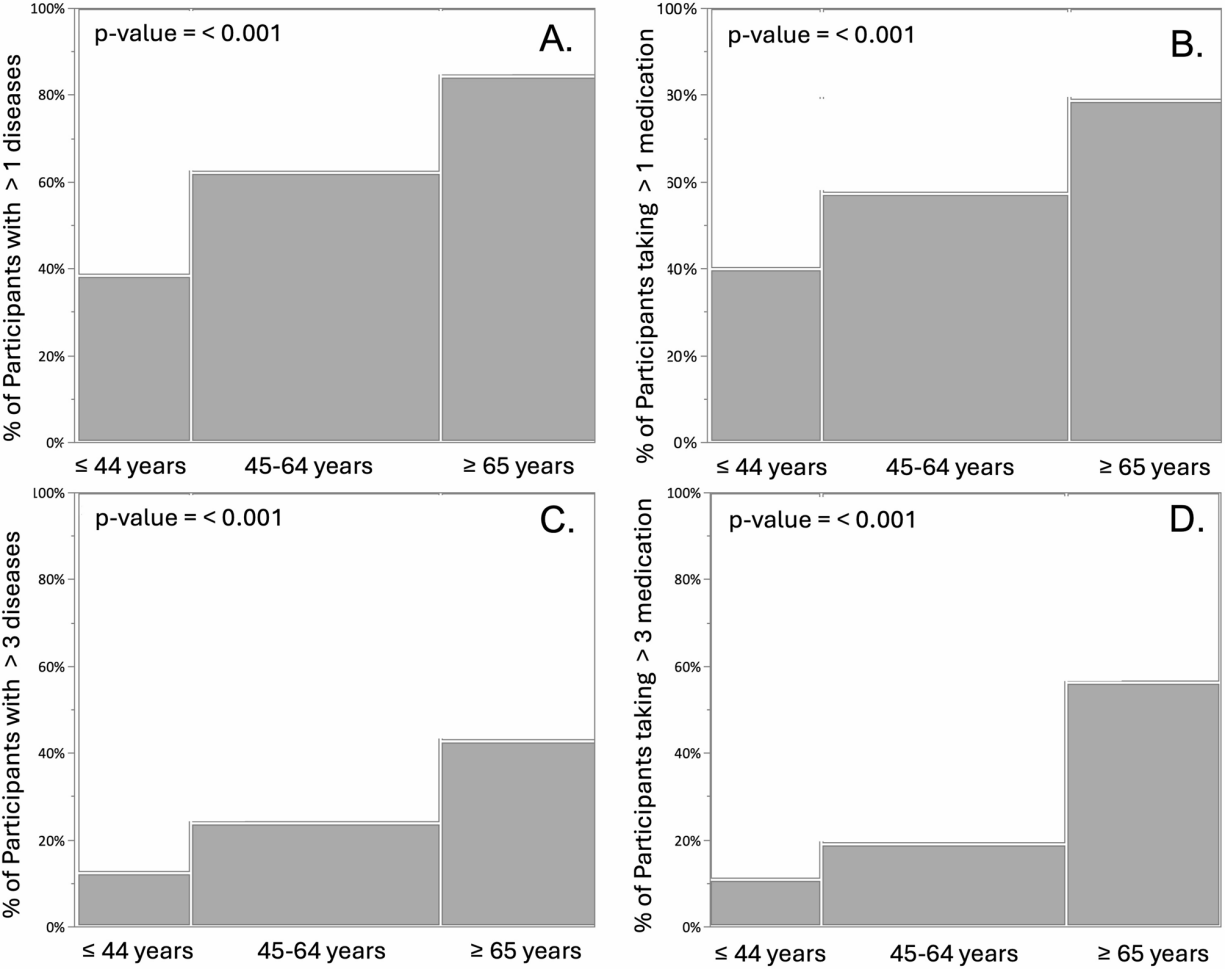
Prevalence of more than 1 and more than 3 diseases and medications across the three age groups

Figure 2 shows the graphical representation of diseases (Fig. 2A and C) and polypharmacy prevalence across the three age groups (Fig. 2B and D).

Using Wilcoxon rank sum test, both results were statistically significant (*p* < 0.05).

### Most common diseases and most frequently taken medications

When analysing specific diseases, hypertension was most prevalent, affecting approximately 33% of all participants. In second place were thyroid-related diseases, which affected 12% of all patients. Several other diseases, such as hypotension, type 2 diabetes mellitus, and asthma, were equally prevalent, each accounting for 8% of all patients in each category. Table 5 shows the 25 most prevalent diseases. In terms of medication intake, angiotensin-converting enzyme inhibitors were in first place (17%), followed by lipid-lowering agents, thyroid hormones, platelet aggregation inhibitors and beta-blockers. Table 6 shows the 25 most prevalent medications.

**Table 5:** Ranking of the 25 most common diagnosed diseases by the participants

Ranking	Disease	No. of patients	%
1	Hypertension	97	32.7
2	Hypothyroidism or thyroid disease	37	12.4
3	Hypotension	24	8.1
4	Type 2 diabetes mellitus	24	8.1
5	Asthma	24	8.1
6	Post malignant neoplasm	14	4.7
7	Rheumatic disease	13	4.4
8	Depression	13	4.4
9	Coagulation dysfunction	13	4.4
10	Osteoporosis	12	4.0
11	Post radiotherapy	11	3.7
12	Post chemotherapy	10	3.4
13	Heartburn	10	3.4
14	Post myocardial infarction	9	3.0
15	Post-stent implantation	8	2.7
16	Hypercholesterolemia	6	2.0
17	Arthrosis	5	1.7
18	Non-specific neoplasm	5	1.7
19	Post-hepatitis	4	1.3
20	Lichen ruber	4	1.3
21	Migraine	3	1.0
22	Multiple sclerosis	3	1.0
23	Tension/cluster headache	3	1.0
24	Panic disorder or panic attacks	2	1.0
25	Crohn’s disease	2	1.0

Number and the percentage value indicate the proportion of patients with this disease in the total population

**Table 6:** Ranking of the 25 most frequently taken medication by the participants

Ranking	Medication	No. of patients	%
1	Angiotensin-converting enzyme inhibitors	49	16.5
2	Lipid-lowering drugs	45	15.2
3	Thyroid hormones	45	15.2
4	Platelet aggregation inhibitors	44	14.8
5	Beta-receptor blockers	36	12.1
6	Oral antidiabetics	25	8.4
7	Proton pump inhibitors	19	6.4
8	Antidepressants	18	6.1
9	Anticoagulants	16	5.4
10	Inhaled bronchospasmo-lytics	13	4.4
11	Antidiabetics (insulins)	12	4.0
12	Glucocorticoids	9	3.0
13	Immunosuppressants	9	3.0
14	Atypical antipsychotics	8	2.7
15	Bisphosphonates	8	2.7
16	Non-steroidal anti-inflammatory/-rheumatic drugs	8	2.7
17	Cytostatics	6	2.0
18	Antipsychotics	5	1.7
19	Uricostatic drugs	5	1.7
20	Sex hormones	5	1.7
21	Alpha-1-adrenoceptor antagonists	4	1.3
22	Anticholinergics	4	1.3
23	Alpha-2 receptor agonists	4	1.3
24	Antianemics	3	1.0
25	Antibiotics	2	0.7

Number and the percentage value indicate the proportion of patients with this medication in the total population

### Allergies and intolerances

A total of 138 patients (46.5%) reported one or more intolerances and allergies. Of these, drug allergies were the most common with *n*=56 patients, while aero allergies (*n*=32) such as animal hair allergies or hay fever share second place in frequency with contact allergies. Women are statistically significantly more likely to suffering from allergies than men (*p* = 0.016). Age had no statistical effect using logistic regression. The overview can be found in Table 7.

**Table 7:** Allergies reported by all participants

Allergies	*N*	%	Documented examples
Drug allergies	56	18.9	Antibiotics, analgesics, local anaesthetics
Aero allergies	32	10.8	Pollen, house dust, animal hair
Contact allergies	32	10.8	Latex, nickel, denture base plastics
Food allergy	12	4.0	Glutamate, stone and pomaceous fruits, nuts
Insect allergies	6	2.0	Bees, wasps, horseflies

Percentages refer to the number of recorded allergies per category relative to the total number of patients; multiple entries per patient possible

A total of 122 patients (41.1%) reported consuming one (*n* = 93; 76.2%) or multiple (*n* = 29; 23.8%) addictive substances. The remaining 175 patients (58.9%) either denied any substance use or provided no information on the matter. Among the users, 76 patients (23.3%) consumed tobacco, 73 patients (22.4%) alcohol, and 2 patients (0.6%) unspecified illicit drugs. Regarding the amount of tobacco consumed, among the 76 smoking patients, 35 (46.1%) reported smoking up to ten cigarettes per day, while 39 (51.3%) reported smoking more than ten cigarettes daily. Two smokers (2.6%) did not provide information on their consumption. As for alcohol consumption, among the 73 patients who reported drinking, 57 (78.1%) stated they drink alcohol rarely, and 9 (12.3%) stated they drink frequently. Seven of the drinkers (9.6%) did not provide any information on their alcohol consumption.

## Discussion

In this study, participants with general diseases clearly outnumbered healthy participants by 64% to 36% with 27% of all participants suffering from three or more diseases. The average age of participants with health conditions was 54 years, with a positive association between age and the number of diseases. This elevated mean age of patients reflects the demographic data of Germany. The proportion of people < 35yrs old in Germany was 51% in 1970, falling to 37% in 2021. Within the same time period, the proportion of people aged 35 to 64yrs rose from 35% to 41%, whereby the proportion of people >65yrs increased from 14% to 22%, and is even expected to reach 28% in 2024 [42]. On their website “7 years longer”, German insurers give reasons for the significant increase in life expectancy and list medical progress as the main reason alongside increasing prosperity, working conditions, lifestyle and level of education [43]. Medical progress is particularly evident in the fact that new medical and pharmacological successes are constantly being achieved in the treatment of specific diseases, the development of medicines and the provision of new vaccines. This does not mean that people are cured of diseases. New medications or surgical techniques mean that patients can continue to live despite their illnesses, but at the same time become increasingly multimorbid and take more and more medication. A good example of this is the use of anticoagulants, which are now used for many cardiovascular diseases, or the use of bisphosphonates and other antiresorptive drug classes. Of course, the question arises as to whether the patients from the student course can be considered representative of the rest of the population.

### Comparison of diseases with other statistics

The five most common diseases in this study were hypertension (32.7%), hypothyroidism (10.4%), and, each at 8.1%, hypotension, diabetes mellitus, and asthma. Medication use increased with age, and 28% of participants were taking at least three drugs. Most were ATC group C medications, followed by A, B, N, H, M, L, and R. This raises the question of whether patients in student dental courses are representative of the general population. Despite potential selection bias in a university setting, our findings align with national data from major German health insurers.

In 2021, Techniker Krankenkasse reported ATC group C drugs as most prescribed, followed by A, N, H, G, M, and B [44]. BKK data showed a similar ranking: C, A, N, H, B, G, R, M. Barmer listed group C highest, followed by M, A, H, N, with R, J, and B at the bottom [45]. Moreover, the Barmer health report for 2021, ranked drugs from group C at the very top, followed at some distance by drugs from the groups M, A, H and N. At the bottom of the list by a wide margin are the groups R, J and finally B [46]. These results largely match our findings, except for groups G and B. Women under 25 receive nearly three times more daily medications than men, likely due to contraceptives (group G), a group underrepresented in our study. Conversely, older patients were overrepresented, explaining higher use of group B drugs. While general morbidity data is scarce, ATC categories reflect disease patterns: cardiovascular conditions (group C) are most prevalent, followed by endocrine and metabolic diseases (group A), suggesting our sample may reflect the broader population.

### Comparison of medication use with other statistics

In this study 28% of all participants (mean age = 62.4 ± 12.2 yrs) took 3 or more medications. For all participants, the use of medication was 1.9. A higher number was found by Heft and Mariotti in their study, in which 40% of seniors took an average of at least three medications, drug consumption was 1.7 per patient [47]. Jainkittivong et al. also found an average medication intake of 1.5 per patient in 510 patients ≥ 60 years treated in a dental clinic. In their study, the four most frequently mentioned drugs were cardiovascular drugs (32%), endocrinological drugs (14.5%), nutritional drugs (12.9%) and drugs that affect the musculoskeletal system (11.4%) [48]. Rhodus et al. conducted a study at dental schools both in 1976 and 1986, they recorded a significant increase in this patient group from 7.3% in 1976 to 24.6% in 1986 and concluded that the number of patients with medical problems at dental schools will steadily increase [49]. In their study, Radfar and Suresh analysed the mean age of the 1,041 patients examined (ratio of men to women = 1:1.2) as 52 ± 18 years, with 54% of all patients having one or more disorders and 53% taking at least one medication. For the authors, the results of their study highlight the medical complexity of the ageing population, which the dental curriculum must address in terms of treatment management [50].

### Treating multimorbid and polypharmacy patients

A total of 63.6% of the patients in this study had at least one diagnosed disease, and 60.1% were taking at least one medication; accordingly, the corresponding values are higher, with an almost equal distribution between sexes. The subsequent implementation in these patients is far more complex, as the treatment measure must be considered from a holistic perspective. This means that pharmacological, internal, psychological and microbiological aspects of each patient must be taken into account. Dental education as an overall concept must in turn focus more strongly on this growing proportion of patients to be able to treat them safely and conscientiously. Medical history interviews and special precautions prior to treatment are becoming even more important. This applies both to basic theoretical training in subjects such as pharmacology, internal medicine or dermatology and to practical therapeutic measures in dental treatment itself, such as medication interactions with local anaesthesia, subgingival periodontal measures during chemotherapy, or anticoagulant therapy with prolonged local bleeding.

### Adaptations to the curriculum

Every medical educational curriculum must be constantly scrutinized by those responsible and the goals and objectives must be regularly reviewed and possibly redefined [51]. Thomas et al. continue that the outcomes of medical education must always be aligned with the “current and future needs” of healthcare and the population and adapted accordingly [51]. Mahnke et al. also point out that older patients in particular, whose concentration and memory performance are slowly declining, may omit illnesses from their anamnesis or no longer know why they need to take a certain medication if the disease occurred a long time ago [52]. For this reason, not only is the medication information often not detailed enough, but also because many patients do not have the necessary medical knowledge to provide specific anamnesis information. This also includes the often-non-specific information about heart problems or the HbA1c value.

Dental treatment of patients increasingly requires a more detailed assessment of their state of health so that treatment can be adapted if necessary. It is therefore necessary to integrate general medicine even more strongly into the dental curriculum and to expand it further. This is despite the fact that the new types of dental therapy to be taught (CAD/CAM procedures, rotary root canal preparation or vertical condensation filling techniques, etc.) will undoubtedly take up more and more space in the education programs. In particular, this implies that the content of the subjects of emergency management, pharmacology and internal medicine must be taught much earlier in the curriculum and more comprehensively in the curriculum. Fortunately, the new Dental Licensing Regulations, which came into force in 2021, already addressed these topics. There are not only the new courses ‘Emergency Medicine’ and ‘Medicine and Dentistry of Ageing and the Elderly’, but also ‘Oral Medicine and Systemic Aspects’, in which the impact of systemic diseases on oral health will also be taught [23].

In their article, Al-Nawas and Grötz explicitly address the demographic change towards an older population and its influence on dental treatment. They also lament the lack of suitable screening aids to make it easier to identify patients at risk. In their opinion, the now close intertwining of dentistry and general medicine is also problematic because the influence of specific and constantly newly developed medications on oral medicine cannot usually be adequately assessed. Ultimately, both undergraduate and postgraduate training must be improved and the additional work that is often required for these patients must be better remunerated in order to avoid inadequate care in the future [53].

In recent years, dental training institutes, dental societies and associations have developed postgraduate curricula for geriatric dentistry, which are increasingly in demand. In addition, most dental associations now have specialist consultants for geriatric dentistry. Furthermore, for the first time in undergraduate studies, the new licensing regulations for dentists include completely new cross-sectional subjects such as “Medicine and Dentistry for the Elderly” and “Emergency Medicine”. Naturally, the supervisors in the patient courses should also be included in these training programs, essentially a form of postgraduate training, as they bear an ever-increasing responsibility. Younger dentists in particular, who have just obtained their license to practise, are often assigned to these courses, not least because of the new supervision ratio in the licensing regulations. They need to receive further training, not only in the treatment concepts of the respective specialty, but also in medical emergency management and risk assessment.

For the further development of the undergraduate curriculum, this means that, on the one hand, students’ knowledge of how to recognise emergencies and primary emergency measures must be improved, for instance through lectures and seminars in internal medicine [27, 54]. On the other hand, students in the clinical section in particular must learn basic practical skills for particularly important emergency measures at the highest level of competence according to Miller, namely ‘can do’ [55]. They have to practise these measures repeatedly in the curriculum, either on dummies or on each other. This practical training must include different levels of invasiveness. Every student must be able to correctly measure blood pressure and blood oxygen levels on patients using suitable equipment, The management of vasovagal syncope should also not be a problem for clinical students, nor should the early recognition and correction of hypoglycaemia through the administration of glucose. Training must be intensified for the latter in particular, because if it is not possible to give sugar by mouth, students must be able to safely access the patient’s vein. In any case, the practical training must be organised in such a way that the patient is adequately cared for until the emergency doctor arrives. Of course, suitable assessment formats must also be identified for the competencies taught, with a particular focus on evaluating practical skills. Notable examples include OSCEs, digital simulations, mini-CEX, and case-based modules. When selecting these formats, the size of the respective department and available human and spatial resources must always be taken into account. In this context, an emergency OSCE would represent an ideal assessment tool [56–60]. All curricular changes should be accompanied by appropriate evaluations [61–63]. These should primarily focus on undergraduate education but must also consider postgraduate training—especially in the case of continuing education for supervising dentists. The latter could also be critically assessed within team or departmental meetings.

As mentioned above, gerodontology in particular should be given much greater consideration in the curriculum. It is not only internal diseases that increase with age, neurological or orthopaedic impairments such as walking can cause considerable problems for patients, or the fine motor skills to hold and use a toothbrush correctly can be lost. Visits to care facilities during the curriculum would be very helpful, perhaps also with simple diagnostic and prophylactic measures for patients with complex morbidity or dementia. For example, the use of age simulation suits to simulate the life situation of an elderly person has been criticised in studies, but can certainly be useful for an empathic and educational effect [64–66]. Finally, the German national catalogue of learning objectives for the dental curriculum is currently being revised. So, if it has not already been done, it would be highly desirable to include this topic in detail in this catalogue as well as a more precise definition of emergency medical care as both theoretical and practical learning objectives [67].

### Limitations

This study has several limitations that should be addressed.

1. The sample stems exclusively from one single dental school and comprises only patients treated in undergraduate courses, which might limit the generalizability of the findings to other institutions or regions, or treatment settings. Nevertheless, the alignment of our results with national health insurance data suggests that key trends in morbidity and medication use are comparable.
2. The study focused on self-reported medical histories, which could lead to memory bias, especially in older patients, who may not remember specific illnesses or medications despite guided inquiries.
3. It should be borne in mind that a university dental clinic may attract a typical patient population due to structural factors. For example, retired or older patients might have greater scheduling flexibility in context of prolonged treatment sessions, as typical for student courses. Moreover, patients suffering from complex medical conditions or polypharmacy may find it difficult to be treated in general dental practices. These factors could contribute to a higher proportion of elderly and medically compromised patients in our cohort, even though the data is comparable to German insurance data.
4. Government recommendations and legal regulations during the COVID-19 pandemic may have influenced patient selection. Elderly and multimorbid patients were advised to postpone elective procedures and avoid non-urgent treatments, such as dental pain management. Public health guidelines also discouraged large gatherings, possibly deterring treatment in open clinical settings with multiple providers, supervisors, and patients. Additionally, travel—especially for older patients—was often difficult or not feasible, as public transport use was to be minimized. The mandatory use of face masks may also have discouraged attendance, while for phobic patients who felt secure only when masked, the requirement to keep the mouth open during treatment may have been a barrier.
5. As this was a descriptive, exploratory cross-sectional study based on a complete sample within a fixed time frame, no sensitivity analyses were conducted.

Hence, future studies should consider a multi-centre design, incorporating longitudinal observations to further evaluate the structure and medical complexity of dental school patient populations. Potential confounding factors such as socioeconomic status, education level, and ethnicity were not recorded in the context of this study and were therefore not included in the discussion or further analysis. Future studies could consider incorporating and addressing these parameters.

## Conclusions

The results of this study demonstrate the medical complexity of the patients treated in the dental school observed. There was a high prevalence of systemic diseases and medication use, both associated with increasing patient age, which was underlined by a significant correlation between age and the burden of disease and medication alike. These findings strongly suggest that undergraduates should receive a comparable or even equivalent foundational training in medical subjects as medical students. This will ensure that future dentists are able to manage systemic health risks in their patients and play a greater role in preventive medicine in general. This equally applies to postgraduate education, which must regularly incorporate not only practical aspects but also, in view of ongoing medical advances, theoretical components (e.g., the impact of novel medications, with bisphosphonates being a prime example), as well as interdisciplinary collaboration—for instance, with nursing professionals or geriatric centers. Therefore, with the limitations of this study, it could be recommended that comprehensive medical education be integrated into dental curricula. Further studies should assess the impact of integrated medical-dental curricula on student competency in managing medically complex patients.

## Supplementary Information

Supplementary Material 1.

Supplementary Material 2.

Supplementary Material 3.

## Data Availability

The datasets generated and/or analysed during this study are available from the corresponding author upon reasonable request.

## Abbreviations

ICD: International Classification of Diseases
ATC: Anatomical Therapeutic Chemical
STROBE: Strengthening the reporting of observational studies in epidemiology
HIV: Human Immunodeficiency Virus
OR: Odds ratio
HbA1c: Glycated haemoglobin
CAD/CAM: Computer-aided design/computer-aided manufacturing
