# Supplementary material for: Mapping the medical status of patients in a dental school: adapting dental curricula to demographic change - a cross-sectional study
Source: BMC Med Educ. 2025 Nov 6;25:1554. doi: 10.1186/s12909-025-08180-w (PMC12590837; doi:10.1186/s12909-025-08180-w)
Supplement: Supplementary file 1 — Supplementary Material 1. [file 12909_2025_8180_MOESM1_ESM.docx]

**S-Table 1:** Logistic regression results for associations between age and systemic diseasss classified by ICD10 codes.

| **ICD-10 Code** | **Chapter Name** | **OR (95% CI)** | **p-Value** | **AICc** | **BIC** | **McFadden R²** |
| --- | --- | --- | --- | --- | --- | --- |
| **IX** | Circulatory system | 1.07 (1.054–1.100) | <0.001 | 345.5 | 352.8 | 0.16 |
| **IV** | Endocrine, nutritional and metabolic diseases | 1.04 (1.023–1.065) | <0.001 | 325.8 | 333.1 | 59 |
| **XXI** | Factors influencing health status | 1.07 (1.038–1.107) | <0.001 | 203.0 | 210.4 | 109 |
| **XIII** | Musculoskeletal system | 1.04 (0.979–1.064) | 0.008 | 177.0 | 184.4 | 44 |
| **X** | Respiratory system | 1.02 (0.991–1.045) | 0.18 | 200.9 | 208.2 | 0.01 |
| **V** | Mental and behavioural disorders | 0.97 (0.945–0.999) | 0.049 | 152.0 | 159.3 | 25 |
| **XI** | Digestive system | 1.02 (0.986–1.056) | 0.24 | 138.4 | 145.7 | 11 |
| **II** | Neoplasms | 1.04 (1.006–1.087) | 0.019 | 144.3 | 151.6 | 43 |
| **III** | Blood and immune disorders | 1.03 (0.997–1.077) | 0.07 | 124.9 | 132.3 | 0.03 |
| **VI** | Nervous system | 1.01 (0.977–1.056) | 0.42 | 110.1 | 117.5 | 6 |
| **XVIII** | Symptoms and signs not elsewhere classified | 1.02 (0.979–1.064) | 0.32 | 103.5 | 110.8 | 0.01 |
| **XIV** | Genitourinary system | 1.03 (0.973–1.088) | 0.31 | 69.2 | 76.6 | 17 |
| **VII** | Eye and adnexa | 1.10 (1.012–1.200) | 0.024 | 55.6 | 63.0 | 122 |
| **XII** | Skin and subcutaneous tissue | 1.02 (0.958–1.088) | 0.52 | 54.3 | 61.7 | 9 |
| Odds ratios (OR) with 95% confidence intervals (CI), p-values, and model fit indices (AICc, BIC, McFadden’s R²) are reported for each ATC category. Significant associations are marked by p < 0.05. | | | | | | |
