# Supplementary material for: Mapping the medical status of patients in a dental school: adapting dental curricula to demographic change - a cross-sectional study
Source: BMC Med Educ. 2025 Nov 6;25:1554. doi: 10.1186/s12909-025-08180-w (PMC12590837; doi:10.1186/s12909-025-08180-w)
Supplement: Supplementary file 2 — Supplementary Material 2. [file 12909_2025_8180_MOESM2_ESM.docx]

**S-Table 2:** Systemic diseases and medications with statistically significant sex differences in prevalence among dental patients.

| **Category** | **n Female (%)** | **n Male (%)** | **p-value** | **Cramér’s V** |
| --- | --- | --- | --- | --- |
| **Endocrine diseases (ICD IV)** | 57 (68.0%) | 27 (32.0%) | 0.02 | 0.13 |
| **Respiratory diseases (ICD X)** | 22 (64.7%) | 12 (35.3%) | 49 | 0.11 |
| **Syst. hormonal medications (ATC H)** | 37 (68.5%) | 17 (31.5%) | 0.0012 | 134 |
