# Supplementary material for: Mapping the medical status of patients in a dental school: adapting dental curricula to demographic change - a cross-sectional study
Source: BMC Med Educ. 2025 Nov 6;25:1554. doi: 10.1186/s12909-025-08180-w (PMC12590837; doi:10.1186/s12909-025-08180-w)
Supplement: Supplementary file 3 — Supplementary Material 3. [file 12909_2025_8180_MOESM3_ESM.docx]

**S-Table 3:** Logistic regression results for associations between age and intake of medication classified by ATC categories.

| **ATC Code** | **Category Name** | **OR (95% CI)** | **p-Value** | **AICc** | **BIC** | **McFadden R²** |
| --- | --- | --- | --- | --- | --- | --- |
| **C** | Cardiovascular system | 1.09 (1.065–1.121) | <0.001 | 303.7 | 311.0 | 193 |
| **A** | Alimentary tract and metabolism | 1.04 (1.016–1.063) | <0.001 | 277.3 | 284.6 | 109 |
| **B** | Blood and blood forming organs | 1.10 (1.063–1.130) | <0.001 | 258.7 | 266.0 | 0.1 |
| **H** | Hormonal preparations (excl. sex hormones and insulin) | 1.03 (1.004–1.049) | 0.016 | 234.3 | 241.6 | 58 |
| **M** | Musculoskeletal system | 1.05 (1.013–1.080) | 0.005 | 176.1 | 183.4 | 44 |
| **N** | Nervous system | 0.99 (0.976–1.019) | 0.83 | 151.2 | 158.6 | 6 |
| **L** | Antineoplastic and immuno-modulating agents | 1.00 (0.969–1.036) | 0.91 | 110.1 | 117.5 | 6 |
| **R** | Respiratory system | 1.02 (0.984–1.055) | 0.28 | 200.9 | 208.2 | 0.01 |
| **G** | Genito urinary system and sex hormones | 1.02 (0.981–1.059) | 0.31 | 69.2 | 76.6 | 17 |
| **J** | Anti-infectives for systemic use | 0.94 (0.871–1.010) | 0.09 | 138.4 | 145.7 | 11 |
| **S** | Sensory organs | 1.07 (0.989–1.164) | 0.09 | 55.6 | 63.0 | 122 |
| Odds ratios (OR) with 95% confidence intervals (CI), p-values, and model fit indices (AICc, BIC, McFadden’s R²) are reported for each ATC category. Significant associations are marked by p < 0.05. | | | | | | |
